# Effects of Cell Seeding Density, Extracellular Matrix Composition, and Geometry on Yes-Associated Protein Translocation in Corneal Fibroblasts

**DOI:** 10.3390/ijms26031183

**Published:** 2025-01-29

**Authors:** Divya Subramanian, Nathaniel S. Tjahjono, Satweka Nammi, Miguel Miron-Mendoza, Victor D. Varner, W. Matthew Petroll, David W. Schmidtke

**Affiliations:** 1 Department of Bioengineering, University of Texas at Dallas, Richardson, TX 75080, USA; divya.subramanian@utdallas.edu (D.S.); nathaniel.tjahjono@utdallas.edu (N.S.T.); satweka.nammi@utdallas.edu (S.N.); vdv@utdallas.edu (V.D.V.); 2 Department of Ophthalmology, University of Texas Southwestern Medical Center, Dallas, TX 75390, USA; miguel.miron@utsouthwestern.edu (M.M.-M.); matthew.petroll@utsouthwestern.edu (W.M.P.); 3 Department of Biomedical Engineering, University of Texas Southwestern Medical Center, Dallas, TX 75390, USA

**Keywords:** cornea, YAP, collagen fibrils, topography, corneal fibroblasts, micropatterns, confinement

## Abstract

Corneal fibroblasts are central to normal and abnormal wound healing in the cornea. During the wound healing process, several biochemical and biophysical signals that are present in the extracellular matrix (ECM) play critical roles in regulating corneal fibroblast behavior. The translocation and activation of Yes-associated protein (YAP)—a main transcriptional factor in the Hippo signaling pathway—is one example of mechanotransduction involving these signals. However, how corneal fibroblasts integrate these simultaneous cues is unknown. In this study, we utilized well-defined micropatterns of aligned collagen fibrils and other ECM proteins to explore the effects of cell density, topography, geometric confinement, and ECM composition on the translocation of YAP in corneal fibroblasts. We observed that when human corneal fibroblasts (HTKs) were confined to narrow micropatterns (50 μm and 100 μm) of proteins, there was a high degree of cell alignment irrespective of cell seeding density. However, the location of YAP was dependent upon the cell seeding density, ECM composition, and topography. YAP was more nuclear-localized on substrates coated with aligned collagen fibrils or fibronectin as compared to substrates coated with monomeric collagen, random collagen fibrils, or poly-L-Lysine. In addition, we also observed that YAP nuclear localization was significantly reduced when HTKs were cultured on aligned collagen fibrils, monomeric collagen, or fibronectin in the presence of monoclonal blocking antibodies against α_5_ or β_1_ integrin subunits. Finally, we observed that HTK cells formed fibrillar fibronectin on both monomeric collagen and aligned collagen fibrils. These findings provide new insights into how simultaneous biochemical and biophysical cues affect YAP localization in corneal fibroblasts.

## 1. Introduction

Cells in the corneal stroma are exposed to a plethora of biochemical and biophysical cues and actively sense changes in extracellular matrix (ECM) composition, elasticity, and topography, as well as their connectivity to neighboring cells. In the uninjured cornea, the stroma is populated by quiescent dendritic cells, called keratocytes, that are nestled between the lamellae of aligned collagen fibrils. These lamellae vary in thickness (0.2–2.5 μm) and width (5–300 μm) [1,2]. The keratocytes form a dense three-dimensional network of interconnected cells via protrusions that communicate with one another via gap junctions [3,4,5]. During corneal injury, the interconnections between the keratocytes are frequently disrupted, and a variety of soluble growth factors such as transforming growth factor-beta (TGF-β), fibroblast growth factor (FGF), and platelet-derived growth factor-BB (PDGF-BB), among others, are released into the wounded area [6]. In addition to alterations to soluble cues, there are changes in the composition of the corneal ECM (e.g., increased fibronectin, tenascin, and type III collagen) and the alignment of the collagen fibrils during wound healing [7,8]. These changes to the local microenvironment cause unactivated keratocytes to differentiate into fibroblasts (repair phenotype) or myofibroblasts (contractile phenotype) [9]. Thus, cell–cell interactions, the composition, and arrangement of ECM components play important roles in the morphology and function of corneal stromal cells.

The majority of the native cornea stroma is made up of type I collagen fibrils, and the unique organization of the aligned fibrils into lamellae is critical to the clarity and shape of the cornea [10,11]. In addition to providing topographical cues, fibrillar collagen has binding sites for interactions with a wide variety of molecules [12], such as glycosaminoglycans [13], proteoglycans [14], and adhesive glycoproteins [15,16,17]. For example, both fibronectin [16] and tenascin [17] have been reported to bind to fibrillar collagen and are present in the corneal stroma following wound injury. Fibronectin has been reported to enhance keratocyte migration while tenascin reduces adhesion and migration [18]. Thus, due to this complex microenvironment, keratocytes normally experience an assortment of simultaneous biophysical and biochemical stimuli such as nanoscale topography from the fibrils, geometric confinement by being situated between lamellae, and different ECM components that mediate bi-directional signaling and mechanotransduction through integrins.

Changes in the composition of the corneal ECM will also lead to changes in cell behavior via new ECM–integrin interactions. In the normal corneal stroma, quiescent keratocytes normally express a number of integrins that bind to collagen and/or fibronectin, such as α_v_β_3_, α_2_β_1_, α_3_β_1_, and α_6_β_1_ [19,20]. Upon injury, or exposure to serum, keratocytes transform into corneal fibroblasts and exhibit alterations to the distribution and expression of their integrins. For example, corneal fibroblasts express α_4_β_1_ and α_5_β_1_ integrins in addition to expressing the same integrins as unactivated keratocytes [20]. Besides mediating attachment to ECM proteins, integrin binding by keratocytes serves several functions such as providing a synergistic interaction with TGF-β/PDGF-BB growth factor signaling in myofibroblast differentiation [21], the maintenance of corneal structural integrity [22], and transducing mechanical signals to the nucleus to differentially regulate various adhesion-dependent signaling pathways [21,23].

Several recent studies have shown that the transcriptional regulator Yes-associated protein (YAP) lies at the center of a number of signaling pathways (e.g., Hippo, Wnt/β-catenin, MAPK, G protein-coupled receptor signaling (GPCR), Notch, and TGF-β/SMAD) and influences the downstream expression of a variety of genes and proteins [24]. Normally, YAP is distributed in both the nucleus and cytoplasm. However, its distribution is not static, and when activated, YAP rapidly translocates from the cytoplasm to the nucleus. The mechanisms and processes that regulate the activity of YAP are diverse and include cell–cell contact [25], cell polarity [26], metabolism [27], topography [28,29], and mechanical cues [30,31]. Despite the clear role of YAP signaling in ocular disease [32,33], corneal epithelial cell contact guidance [34], regeneration [35], and chronic inflammation [36], only a few studies have examined YAP signaling in corneal keratocytes or fibroblasts [37]. Furthermore, how biophysical cues such as topography, geometric confinement, cell density, and ECM composition regulate YAP translocation in corneal fibroblasts remains unclear.

Micropatterning techniques have proven to be versatile and important for studying and manipulating a variety of cell behaviors. Through the creation of microenvironments with well-defined topographical, geometrical, compositional, mechanical, and/or adhesive cues, a number of cell behaviors can be influenced such as cell activation, differentiation, migration, orientation, and proliferation. In particular, the use of geometric micropatterns can significantly impact cell behavior and signaling pathways by regulating cell–cell contacts and cell spreading or size. For example, it has been shown that micropatterns that control cell size and cell–cell contacts can influence osteogenic differentiation [38], the differentiation of [39] bone mesenchymal stem cells [40], gene expression patterns in fibroblasts [41], sarcomere development in stem cell-derived cardiomyocytes [42], and the contractile strength of vascular smooth muscle myocytes [43].

Previously, we reported a microfluidic patterning technique that deposits aligned collagen fibrils on hydrophobic glass and PDMS substrates that possess a similar organization and structure of type I collagen fibrils as the native corneal stroma. With these substrates, we were able to assess the effects of aligned fibril topography on cell alignment and spreading area [44,45,46]. In this study, we determined the effects of biophysical (topography, geometry, and cell density) and biochemical (different ECM proteins) cues on mechanotransduction in corneal fibroblasts. Cells were cultured on micropatterns and ECM protein coatings of different geometry, and we observed that changes in cell alignment and YAP localization were dependent on the micropattern geometry, cell density, and ECM composition. Finally, we investigated the role of different integrin subunits on YAP nucleo-cytoplasmic localization through integrin-blocking experiments. By examining the behavior of corneal fibroblasts on micropatterns of different geometries, ECM coatings, topographies, and confinements, we illustrate a hierarchy of cues regulating corneal cell behavior.

## 2. Results

### 2.1. Geometric Confinement and Cell Density Modulate Nuclear Alignment and Area

Previous experiments with HTK cells cultured on confined micropatterns of aligned collagen fibrils showed that cell alignment increases as the width decreases [46]. To investigate whether geometric confinement was a more powerful cue than topography in promoting cell alignment, we fabricated micropatterns of monomeric collagen, fibronectin, and aligned collagen fibrils of identical widths on PDMS-coated glass coverslips with an anti-adhesive Pluronic coating surrounding them (Supplementary Figure S1). The monomeric collagen micropattern allowed for a direct comparison of the topography of the aligned collagen fibrils with the same ECM type (i.e., type I collagen), while the fibronectin micropattern allowed us to investigate how different ECM types might influence the role of geometric confinement. The fluorescent images of monomeric collagen and fibronectin micropatterns in Supplementary Figure S1 demonstrate that high-fidelity patterns and uniform protein coatings were obtained over the different micropattern widths (50–750 µm) (Figure S1).

**Figure 1:**
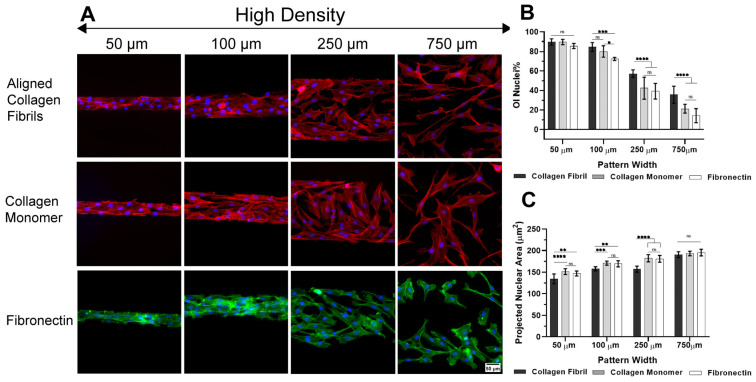
Effects of geometric confinement on HTK cell alignment and nuclear area at high cell seeding density. (**A**) Representative fluorescent images of HTKs cultured on micropatterns of aligned fibrils, collagen monomer, and fibronectin patterns at high cell seeding density. Top and middle panel: phalloidin (red) and DAPI (blue). Bottom panel: phalloidin (green) and DAPI (blue) (**B**) Plot of orientation index (OI) and (**C**) projected nuclear area for HTKs cultured on the different protein micropatterns. Data represent mean ± standard deviation over 4 repeats. Scale Bar = 50 μm. ns p > 0.05; * p < 0.05; ** p < 0.01; *** p < 0.001; **** p < 0.0001.

When HTKs were cultured on substrates containing micropatterns of monomeric collagen and fibronectin surrounded by Pluronic at 15,000 cells/mL in basal media, we observed similar cell behavior to that observed when the cells were confined to micropatterns of aligned collagen fibrils (Figure 1A). Cells were densely packed on the narrow patterns (50 µm and 100 µm) and filled the entire protein micropattern areas, while on the 250 µm and 750 µm wide patterns, cell packing was reduced. To quantify the degree of cell alignment on the different micropattern coatings, we measured the nuclear orientation index (OI) (Figure 1B). On the 50 µm wide micropatterns, the OI values were greater than 80% for all of the protein coating types, with no significant difference between the groups. However, as the width of the micropatterns increased, the degree of alignment depended upon the type of protein coating. When the micropattern width was increased to 100 µm, there was still no difference between the OI values for the monomeric and aligned collagen fibril micropatterns. However, the OI value for the 100 µm wide fibronectin micropattern was significantly smaller than the monomeric or aligned collagen fibril patterns. Conversely, on the 250 µm and 750 µm wide micropatterns, there was no significant difference between the OI nucleus values for the collagen monomer and fibronectin patterns, but the OI value for the aligned collagen fibril micropattern was significantly higher. These results suggest that when cells are densely packed on narrower micropatterns (50 μm and 100 μm), geometric confinement is a stronger cue than topography, and that confinement promotes cell alignment independent of the protein coating. Conversely, when cells have more space to spread and move on the larger micropatterns, the topography of the aligned collagen fibrils becomes a more important cue for promoting an aligned cellular orientation.

In addition to nuclear alignment, we also measured the size of the projected nuclear area and observed that the size of the nuclear area decreased as the width of the geometric confinement decreased (Figure 1C). On the largest micropatterns (~750 µm), the projected nuclear area was ~200 µm^2^ and was independent of the type of ECM coating. However, as the width of the micropattern decreased, the nuclear area also decreased with a nuclear area of ~150 µm^2^ on the 50 µm wide micropatterns. We also observed that as the width of the micropatterns decreased, the cells cultured on micropatterns that contained both topography and confinement (i.e., collagen fibrils) had statistically smaller nuclear areas than on micropatterns without topography (i.e., monomeric collagen, fibronectin). A possible explanation for this reduction in nuclear area on the smaller micropatterns is that the high cell density progressively forces individual cells into smaller areas, increases compressive forces and cell–cell contacts, and leads to contact inhibition [25,47].

**Figure 2:**
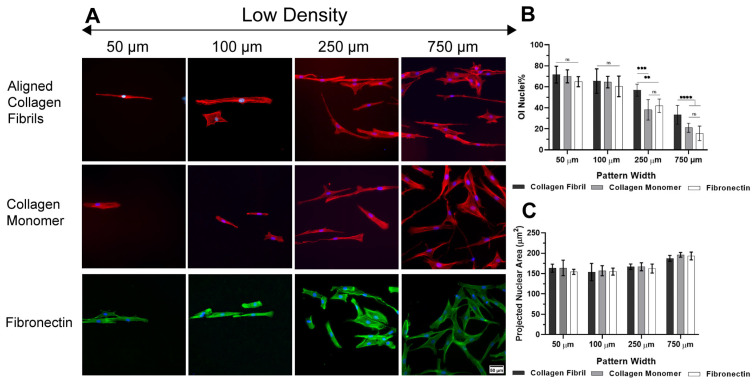
Effects of geometric confinement on HTK cell alignment and nuclear area at low cell seeding density. (**A**) Representative fluorescent images of HTKs cultured on micropatterns of aligned fibrils, collagen monomer, and fibronectin patterns at low cell seeding density. Top and middle panel: phalloidin (red) and DAPI (blue). Bottom panel: phalloidin (green) and DAPI (blue). (**B**) Plot of orientation index (OI) and (**C**) projected nuclear area for HTKs cultured on the different protein micropatterns. HTK cells were seeded at 1000 cells/mL for the 50 μm wide micropatterns, 2000 cells/mL for the 100 μm wide micropatterns, 5000 cells/mL for the 250 μm wide micropatterns, and 15,000cells/mL for the 750 μm wide micropatterns. Data represent mean ± standard deviation over 4 repeats. Scale Bar = 50 μm. ns p > 0.05; p < 0.05; ** p < 0.01; *** p < 0.001; **** p < 0.0001.

To investigate the effect of cell density on alignment and changes in the nuclear area of HTKs, we seeded HTKs at the same cell density/area across the different micropattern widths for aligned collagen fibrils, collagen monomer, and fibronectin (Figure 2A) to minimize cell–cell contact. When HTK cells were cultured at low cell densities, no significant differences in the OI values were observed for the different protein coatings on the narrow micropatterns (50 μm and 100 μm). It should be noted, however, that the OI values of ~70% and ~65% for HTKs cultured at low cell density on the 50 μm and 100 μm micropatterns (Figure 2B) were considerably lower than the OI values (~89%) measured when HTKs were cultured at a high density on the narrow micropatterns (Figure 1B). In addition, when culturing HTKs at lower cell densities on the wider micropatterns (250 μm and 750 μm), we once again observed higher OI values on the aligned collagen fibrils compared to the flat monomeric collagen and fibronectin micropatterns.

In contrast to the changes in nuclear area that were observed on the different micropattern widths and ECM coating when HTK cells were seeded at a high cell density, we observed no significant differences in nuclear area when cultured at low cell densities. As shown in Figure 2C, the nuclear area was relatively constant (~165 µm^2^) for the 50–250 μm wide micropatterns, while there was a small non-significant increase in the nuclear area (~195 µm^2^) on the 750 μm wide micropatterns. However, for a given micropattern width, the type of ECM coating did not influence the nuclear area. These results also support the idea that changes in cell–cell contacts and available cell spreading area impact the nucleus and its morphology. Overall, these results indicate that when cells are tightly packed together, geometric confinement is a stronger cue than topography or ECM coating in inducing cell alignment on narrow micropatterns. Yet, when cells are moderately to sparsely packed, the topography of the aligned collagen fibrils serves as a cue to induce cell alignment.

### 2.2. Confinement and ECM Coating Modulate Yes-Associated Protein (YAP) Localization

**Figure 3:**
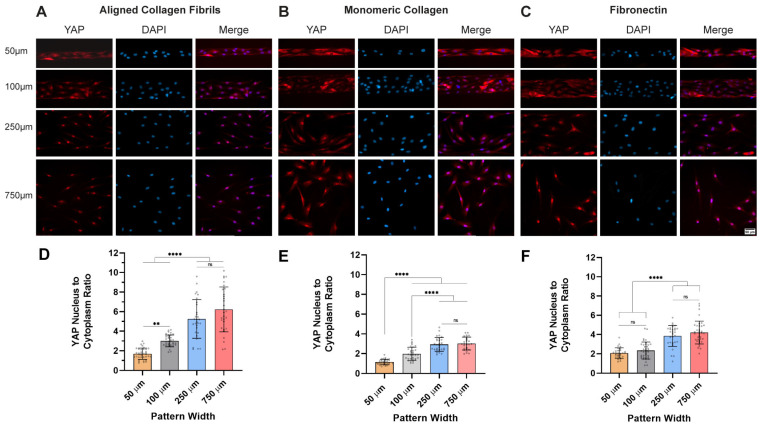
Confinement to micropatterns at high density modulate YAP localization on corneal fibroblasts: representative fluorescent images of HTK cells cultured on micropatterns of (**A**) aligned collagen fibrils, (**B**) monomeric collagen, and (**C**) fibronectin of varying widths of 50 μm, 100 μm, 250 μm, and 750 μm for 24 h, and cells were stained for YAP (Red) and nuclei (Blue). Scale Bar = 50 μm. The nuclear/cytoplasm ratio of YAP was then plotted as a function of the micropattern width for the 3 proteins (**D**–**F**). Data represent mean ± standard deviation over 4 repeats. ns p > 0.05; ** p < 0.01; **** p < 0.0001.

We also investigated how geometric confinement, topography, and cell density individually and collectively impacted YAP localization. When HTK cells were cultured at a high cell density on large micropatterns of aligned collagen fibrils (width = 250 µm and 750 µm), the qualitative examination of YAP-stained fluorescent images revealed that YAP was primarily located in the nucleus on the wide substrates (Figure 3A). In contrast on the narrow micropatterns of aligned collagen fibrils (50 µm and 100 µm), there was a shift in YAP to the cytoplasm (Figure 3A). We observed similar trends in the YAP localization on confined micropatterns of monomeric collagen (Figure 3B) or fibronectin (Figure 3C), although there were some qualitative and quantitative differences in the different ECM coatings.

Next, we quantified the changes in YAP localization by measuring the fluorescent intensity of YAP in the nucleus and the cytoplasm and calculated the nuclear/cytoplasmic (N/C) ratio. For all ECM types, we observed significant differences in the N/C ratio on the narrower and wider patterns (Figure 3D–F). On micropatterns of aligned collagen fibrils (Figure 3D), we observed the N/C ratio of 1.7 on the 50 μm wide micropattern, which increased with the micropattern width, reaching a maximum value of 6.2 on the 750 μm wide micropattern. We observed a similar trend of increasing the N/C ratio with the micropattern width on both the monomeric collagen (Figure 3E) and fibronectin (Figure 3F) micropatterns. It should be noted, however, that the range of N:C ratios on the monomeric collagen micropatterns (1.5 to 3.0) was about 1.5- to 2-fold lower than those observed on the aligned collagen fibrils (1.7 to 6.2) or fibronectin micropatterns (2.1 to 4.2).

Previous studies have shown that cell–cell contact can regulate YAP localization, where YAP is normally localized to the nucleus of cells grown at a low density but translocate to the cytoplasm in confluent or high-density cell cultures [48,49]. Since HTK cells grown on the smallest micropatterns formed a confluent layer after only 24 h of cell culture, we conducted a pilot study to examine how culturing HTK cells at different cell densities would affect the localization of YAP. When HTK cells were grown on 250 μm wide micropatterns of monomeric collagen (Supplementary Figure S2A–C), the N/C ratio increased as the cell density decreased (Figure S2D). These results suggest that contact inhibition may be partially responsible for the changes in YAP localization with micropattern width that were observed in Figure 3 when HTK cells were cultured at a high cell density.

**Figure 4:**
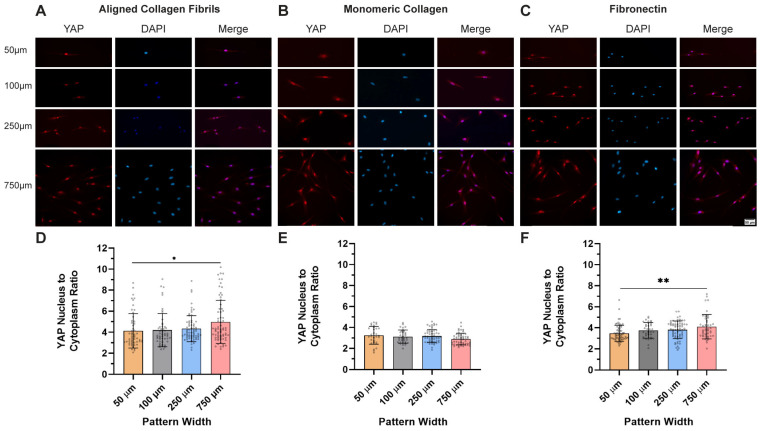
Corneal fibroblasts when cultured at the same cell density on different widths show nuclear YAP localization: confined low-density representative fluorescent images of HTK cells cultured on micropatterns of (**A**) aligned collagen fibrils, (**B**) monomeric collagen, and (**C**) fibronectin of varying widths of 50 μm, 100 μm, 250 μm, and 750 μm for 24 h. Cells were stained for YAP (Red) and nuclei (Blue). Scale Bar = 50 μm. The nuclear/cytoplasm ratio of YAP was then plotted as a function of the micropattern width for the 3 proteins (**D**–**F**). Data represent mean ± standard deviation over 4 repeats. * p < 0.05; ** p < 0.01.

To further investigate the role of cell density on YAP localization on the different micropattern widths, we also cultured HTKs at the same cell density/area across the different widths (Figure 4A–C). Interestingly, on the collagen monomer micropatterns, we observed no significant differences in the YAP N:C ratio (3.0 to 3.2) in HTK cells cultured on the different micropattern widths (Figure 4E), and the spread of the YAP ratios was relatively constant. In contrast, on the aligned collagen fibril micropatterns, the YAP N:C ratio was relatively constant (4.1 to 4.3) for the 50 μm to 250 μm widths but increased significantly to 5.0 on the 750 μm wide micropatterns. It is also noteworthy that the spread of the YAP ratios on the aligned collagen fibrils (Figure 4D) was much greater, with some ratios as high as 10. We observed a similar trend in the YAP N:C ratios on the fibronectin micropatterns (Figure 4F) with a significant difference between the 50 μm (ratio = 3.5) and the 750 μm (ratio = 4.2) widths. It is interesting to note that even at low cell densities, the YAP N:C ratio was different on the aligned collagen fibril, collagen monomer, and fibronectin patterns, which is in agreement with other studies that have reported that YAP localization is dependent upon the ECM [50].

**Figure 5:**
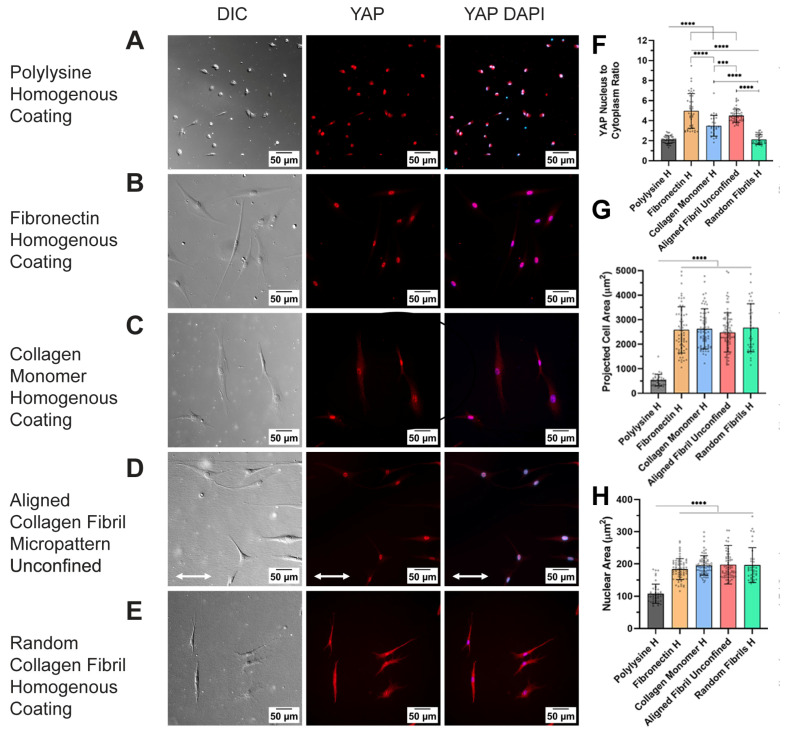
YAP translocation in corneal fibroblasts depends on protein type: representative fluorescent images of HTK cells cultured on homogeneous coatings of (**A**) poly-lysine, (**B**) fibronectin, (**C**) monomeric collagen, (**D**) 750 μm aligned collagen fibrils, and (**E**) random fibrils. Cells were stained for YAP (Red) and nuclei (Blue). Scale Bar = 50 μm. The YAP nuclear/cytoplasm ratio was then plotted as a function of the underlying protein type (**F**). The projected cell (**G**) and nuclear area (**H**) are plotted for each protein type. Data represent mean ± standard deviation over 4 repeats. White double arrow indicates fibril direction, and fibrils are oriented horizontally. *** p < 0.001; **** p < 0.0001.

To further examine the roles of geometric confinement and protein type on YAP localization, we conducted a second set of experiments in which we cultured HTK cells on different protein types but in the absence of confinement. In specific, HTKs were cultured on homogenous coatings of fibronectin, collagen monomer, random fibrillar collagen, and poly-L-lysine (PLL), as well as an unconfined 750 μm wide micropattern of aligned collagen fibrils (Figure 5A–E). We included the poly-lysine (PLL)-coated substrates to determine how the non-specific adhesion of HTKs would impact YAP localization. Likewise, we included the random fibrillar collagen substrate to examine the impact that fibril alignment had on YAP localization, since previous studies with adipose-derived mesenchymal stem cells (ASCs) have reported that aligned fibrous matrices promote YAP/TAZ nuclear translocation and activation [51]. As shown in Figure 5, HTK cells cultured at a low density on the unconfined 750 μm wide aligned collagen fibril micropattern and the homogenous coatings of fibronectin, collagen monomer, and random fibrillar collagen exhibited a spindle morphology. In addition, the HTK cells on these coatings all had similar cell spreading areas (~2500 μm^2^, Figure 5F) and projected nuclear areas (175 μm^2^, Figure 5G). In contrast, when HTK cells were cultured on the PLL-coated substrates, they had a small, rounded morphology with reduced cell spreading and projected nuclear areas.

We also assessed whether culturing HTK cells on homogenous ECM coatings would alter the ECM-induced YAP nuclear translocation results that were observed on the confined micropatterns (Figure 4). Similarly to the results on confined micropatterns, the YAP N:C ratio for HTK cells on the homogenous, unconfined coating of collagen monomer (3.4) was lower than on the aligned collagen fibrils (4.5) coating and the fibronectin coating (5.0) (Figure 5H). It is unclear why the YAP N:C ratio on the homogenous fibronectin coating (5.0) was slightly higher than on the confined fibronectin coating (4.2) and the aligned collagen fibril micropattern (4.5). One possible explanation is that the different coating procedure on the homogenous substrate vs. the micropattern substrate may lead to differences in the amount of fibronectin that adsorbs. On the unconfined PLL coating, the YAP N:C ratio was 2.1, suggesting little nuclear translocation. This result was not surprising given the small, rounded morphology of HTKs on PLL. Surprisingly, the YAP N:C ratio on the random collagen fibrils was also 2.1. This result was not expected given the differences in cell morphology and spreading area on random collagen fibrils versus the PLL coating.

### 2.3. Blocking Specific Integrin Subunits Inactivates YAP in Corneal Fibroblasts

**Figure 6:**
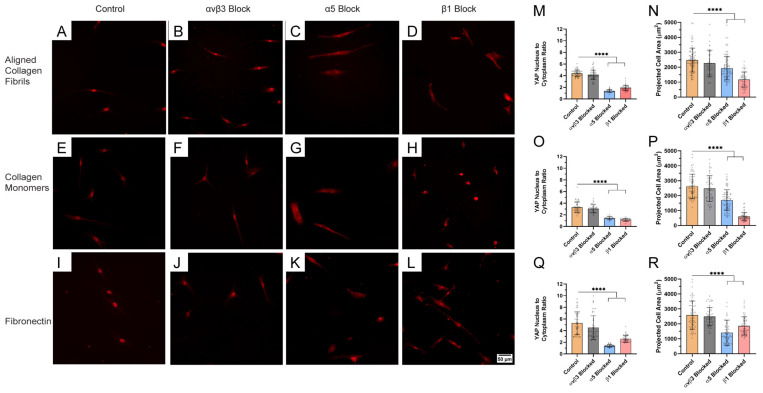
Blocking integrin subunits modulates cell spreading and YAP translocation depending on ECM type: representative fluorescent images of HTK cells cultured on unconfined aligned collagen fibrils (750 μm wide), and homogenous coatings of monomeric collagen and fibronectin for 24 h in the absence and presence of integrin-blocking antibodies (**A**–**L**). Cells were stained for YAP (red). Scale Bar = 50 μm. YAP N/C ratio and cell area are plotted for each ECM type (**M**–**R**). **** p < 0.0001.

Previous studies with other cell types have shown that integrin-mediated signaling is a key determinant of YAP nuclear localization. To determine how and which integrins modulate adhesion and YAP signaling in corneal fibroblasts to different ECM proteins, we cultured HTKs in basal media in the presence of specific integrin-blocking antibodies (Figure 6) and measured their YAP N:C ratios (Supplementary Materials Table S1) as well as their spreading areas (Supplementary Materials Table S2). HTKs cultured in basal media without any function-blocking antibodies were used as controls. We also tested if blocking another known fibronectin integrin αvβ3 modulated YAP translocation [20]. The treatment of HTK cells with an antibody against the α_V_β_3_ integrin, a known fibronectin receptor [20], resulted in no reduction in YAP translocation on the aligned collagen fibril and monomeric collagen substrates, and only a small reduction that was not statistically significant on the fibronectin substrate (Figure 6). Similarly, α_V_β_3_ integrin blocking resulted in small reductions in the cell spreading area on all three protein coatings that were statistically insignificant (Table S2).

As expected, blocking the adhesion of the α_5_ integrin subunit (another fibronectin receptor) significantly inhibited YAP translocation into the nucleus and significantly reduced cell spreading on fibronectin-coated substrates. Surprisingly, we also observed that α_5_ integrin blocking also caused reductions in YAP translocation and cell area on both the monomeric collagen and aligned collagen fibrils substrates. The exact cause for these results on collagen when blocking the α_5_ integrin subunit is unknown; a possible explanation is provided in the Discussion. It should be noted, however, that a similar reduction in YAP levels was observed when bone-marrow-derived human mesenchymal stem cells were treated with a blocking α_5_ integrin antibody and cultured on collagen polyacrylamide-coated hydrogels [50].

When HTK cells were treated with a blocking mAb against the β_1_ integrin subunit (a subunit known to adhere to collagen, fibronectin, and other ECM proteins), both translocation into the nucleus and cell spreading area was significantly reduced on monomeric collagen coatings, suggesting that β1 integrin was the primary integrin responsible for HTKs adhering to monomeric collagen. Blocking of the β_1_ integrin subunit of HTK cells cultured on aligned collagen fibrils also significantly reduced YAP translocation into the nucleus and the cell spreading area as compared to the controls. However, it should be noted that the cell spreading area on aligned collagen fibrils (1096 μm^2^) in the presence of β_1_ integrin subunit blocking was almost twice the cell spreading area (605 μm^2^) on monomeric collagen. While the specific mechanisms for these results have not been explored at this time, differences in integrin engagement by the cells on aligned fibril vs. collagen monomer-coated substrates or differences in ligand presentation and densities for cell attachment might be a probable cause. This result suggests that integrins other than β_1_ integrins may be involved in HTK cell adhesion to aligned collagen fibrils. Finally, the treatment of HTK cells cultured on fibronectin-coated substrates with a blocking mAb against the β_1_ integrin subunit also led to significant reductions in YAP translocation and cell spreading areas as compared to the controls. However, cell spreading on fibronectin (1860 μm^2^) in the presence of the β_1_ integrin subunit blocking mAb was significantly higher than the spreading areas on either of the collagen substrates. Taken together, these results suggest that different integrin subunits are involved in the adhesion of HTK cells to the different ECM proteins which in turn differentially modulate YAP signaling.

### 2.4. Effect of Protein Coating on HTK Cell Fibrillar Fibronectin

**Figure 7:**
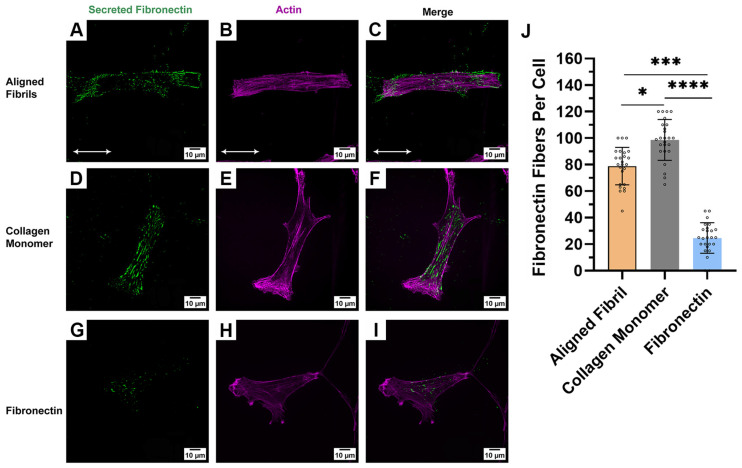
Fibronectin matrix deposition is induced by corneal fibroblasts: immunofluorescence confocal images for fibronectin deposition by the cells on aligned collagen fibrils- (**A**–**C**), monomeric collagen- (**D**–**F**), and fibronectin-coated (**G**–**I**) substrates. Cells were labeled for fibronectin (Green) and actin (Magenta). (J) Quantification of fibrillar fibronectin deposition per cell. White double arrow indicates fibril direction, and fibrils are oriented horizontally (Scale Bar = 10 μm). Total amount of fibronectin fibrillar matrix deposited per cell after 24 h of culture in basal media. Data represent mean ± SD for *n* = 4 repeats for a total of >20 cells. * p < 0.05; *** p < 0.001; **** p < 0.0001.

We aimed to investigate whether the effects of blocking the α5 integrin subunit on collagen-coated substrates might be related to the HTK cells secreting fibronectin when cultured on the different ECM protein coatings. When HTKs were cultured on the three different ECM protein-coated substrates, we observed the deposition of a fibronectin fibrillar matrix on all three substrates (Figure 7). However, the amount of fibronectin deposited by the HTK cells varied with the underlying ECM protein coating. On the collagen-coated substrates (aligned fibrillar and collagen monomer), we observed significant amounts of fibronectin fibrillar matrix deposition (Figure 7A–F), while less fibrillar fibronectin was deposited by HTK cells on the fibronectin-coated substrate (Figure 7G–I). Quantitative analysis of the amount of fibrillar fibronectin deposited by each cell revealed that the highest amount of fibrillar fibronectin deposition occurred on collagen monomer-coated substrates, followed by aligned collagen fibril-coated substrates, and fibronectin-coated substrates (Figure 7J). It should be noted that most cells cultured on the fibronectin-coated substrates did not show any fibronectin fibril deposition. While the amount of deposited fibronectin fibers per cell were analyzed for each cell, not all cells had fibronectin fibers directly below the cell. In the majority of cells, the fibronectin staining was observed in close proximity (<50 μm) to the cell membrane, which suggests that the cells may have laid down the fibronectin and migrated to a new location (Supplementary Figure S3). We also observed that HTK cells adhered to the uncoated PDMS outside of the aligned collagen fibril micropatterns, but we did not observe any fibronectin matrix deposition (Supplementary Figure S4). In addition, no fibronectin staining was observed in absence of cells on aligned collagen substrates. (Supplementary Figure S5). These results suggest that the ability of HTK cells to deposit fibrillar fibronectin is dependent upon the type of ECM coating.

## 3. Discussion

Corneal stroma cells perform two important functions: (i) maintenance of stromal transparency through matrix synthesis and (ii) repair of the extracellular matrix (ECM) following injury. These functions are related to the ability of these cells to sense changes in their microenvironment (e.g., surface topography, ECM composition, communication with neighboring cells) and to alter their behavior (i.e., migration, proliferation, contractility) and transition into repair phenotypes (i.e., fibroblasts or myofibroblasts). Previous studies have shown that corneal keratocytes and fibroblasts are highly sensitive to both biochemical and biophysical stimuli present in the corneal stroma, including growth factors [52,53,54], topography [45,55,56,57], ECM composition [18,58], and matrix stiffness [23,59,60,61]. In addition, prior studies have also shown that cell seeding density can impact (i) whether proteoglycans (e.g., keratocan) secreted by corneal keratocytes cultured in vitro are degraded [62], (ii) the expression levels of transforming growth factor beta (TGF-β) and Smad2 localization in corneal fibroblasts [63], and (iii) whether corneal fibroblasts differentiate into myofibroblasts [64]. While studies in other cell types have shown that YAP signaling is an important component in the cellular response to these biochemical and biophysical stimuli [24,65], knowledge of how these cues impact YAP localization in corneal stromal cells is limited. Understanding the regulation of YAP activity is an important step in characterizing physiological and pathological processes in the cornea. Thus, the purpose of this study was to investigate how different biochemical and biophysical cues influence YAP localization in corneal stroma fibroblasts.

### 3.1. Effects of Cell Density, Confinement, and Topography on Cell Alignment and YAP Localization

When HTK cells were seeded at either low or high cell density on 2D geometric micropatterns of aligned collagen fibrils, monomeric collagen, or fibronectin, the degree of HTK cell alignment increased as the width of the micropattern decreased. This observation was consistent with prior studies reporting that geometric constraints can modulate cell alignment in a number of cell types (e.g., myofibroblasts [66], neonatal rat ventricular myocytes [67], neuroblastoma cells [68], and human-derived MCF-10A breast epithelial cells [69]). On wider stripe patterns (>250 μm), there was an increased degree of cell alignment on the aligned collagen fibril micropatterns, which contained both topography and confinement, than on the micropatterns that only exposed cells to confinement (i.e., monomeric collagen, fibronectin). The observation that the high degree of HTK cell alignment on the 50 μm wide micropatterns was independent of cell density, the type of ECM protein (fibronectin vs. collagen), or topography (fibrillar vs. monomeric collagen) suggests that on narrow patterns (2–4 cells wide), confinement was the primary driver of alignment. This result agrees with the entropy model proposed by Buskermolen et al. [66] which predicts that cells will wander and change shape to maximize their morphological entropy when a protein pattern line is above critical strip width (w_crit_). Conversely, this model predicts that on smaller stripes (<w_crit_), the spatial geometric constraints cause biochemical changes that result in enhanced cell polarization and alignment with the stripe orientation.

Previous studies have shown that Yes-associated protein (YAP) is a nuclear transducer of mechanical signals that mediates various downstream signals relevant to a number of physiological processes such as proliferation, motility, and differentiation [24] that are important in corneal wound healing. For example, YAP has been shown to be a distinct modulator of transforming growth factor β (TGFβ)-induced corneal myofibroblast transformation [37]. Likewise, it has been reported that YAP signaling is important during the regeneration of corneal epithelium in both chronic inflammation [36] and normal wound healing [35]. Although it is well known that substrate stiffness [33] and geometry [70,71] can affect the localization of YAP, the role of topography in YAP localization in corneal cells is not completely understood [34]. We observed that when the HTK cells were seeded at a high cell density on the large micropatterns, the majority of the YAP was located in the nucleus (e.g., collagen monomer ~75%, fibronectin ~81%, 86% collagen fibrils). However, as the level of confinement increased on the narrower micropatterns, there was a movement of YAP from the nucleus into the cytoplasm when cells were highly packed. In contrast, when the cell seeding density was lowered, the YAP N/C ratio was independent of the micropattern width on all three protein coatings. These results are in agreement with other studies that have shown that cell–cell contact inhibition is a strong driver of YAP localization [25,65].

The observation that the YAP N/C ratio was consistently higher on the aligned collagen fibril micropatterns than on the micropatterns of monomeric collagen suggests that topography also plays a role in regulating YAP localization in corneal fibroblasts. These results appear to agree with other studies that have reported changes in YAP localization in cardiac fibroblasts [72,73] and annulus fibrosus-derived stem cells [72,74] due to the topography of the aligned structures. Since nuclear YAP has been observed in migrating cells [73,75] and studies have shown that corneal fibroblasts show directed migration on aligned nanogroove substrates [74,76], our observation of high YAP N/C ratios on aligned fibril substrates might be due to directed migration as compared to other ECM coatings. Alternatively, cellular elongation along the aligned fibril substrates might also cause YAP nuclear entry [75,77].

### 3.2. Effects of ECM Coating and Integrin Blocking on YAP Localization and Fibronectin Fibril Deposition

The composition of the extracellular matrix is known to play a key role in regulating the behavior of cells by providing adhesive biochemical cues, topographical cues, and mechanical signals. Recent studies in human mesenchymal stem cells (hMSCs) have shown that both the type of ECM and the ECM density can influence biochemical ligand-induced YAP translocation [50,76,78]. However, how ECM composition influences YAP translocation in corneal fibroblasts is largely unexplored. Most studies with corneal fibroblasts and composition effects have reported that these cells are sensitive to ECM composition in terms of spreading and migration [18,58]. Our studies on how ECM type influences YAP localization are novel, and the observation that the YAP N/C ratio on the fibronectin micropatterns was higher than the monomeric collagen micropatterns suggests that ECM composition affects YAP localization in corneal fibroblasts. These results are in agreement with reports that adhesion to fibronectin regulates Hippo signaling and YAP nuclear translocation via the FAK-src-PIK pathway [77,79]. It should be noted that a limitation of our study is that we did not examine how the ligand density of the different ECM types influenced YAP localization. The fact that the YAP localization changed but the cell spreading area did not vary on the different ECM coatings might suggest that different ligand densities were not important; however, this needs to be confirmed. A second limitation of this study is that we only example a single ECM protein at a time, while in vivo cornea fibroblasts are exposed to multiple ECM proteins. We did not investigate whether the signaling from one ECM component (i.e., fibronectin) would override the signal from a second ECM component (i.e., collagen) in this study, but we have previously examined the effects of fibronectin coatings on aligned collagen fibrils in the activation of primary rabbit corneal keratocytes [45].

Determining the integrin-mediated adhesion and signaling properties of corneal fibroblasts is complicated due to the large number of distinct integrin heterodimers that are present on the cell surface. Using a series of monoclonal blocking antibodies, we were able to observe how different integrin subunits alter YAP localization. Although prior studies have established that corneal fibroblasts express the α_5_ integrin subunit as a fibronectin receptor [19,78,80], and that α_5_β_1_ is involved in fibronectin assembly into fibrils at the cell surface [79,81], we were surprised to observe that blocking the α_5_ integrin subunit reduced cell spreading and YAP translocation on the collagen substrates (e.g., monomeric collagen and aligned fibrils). To our knowledge, only one previous paper [76,78] has reported that blocking α_5_ integrin reduces cell adhesion and YAP localization on collagen-coated substrates.

One possible explanation of these results is that the HTK cells were secreting and depositing their own fibronectin onto the collagen substrates, and that HTK cell adhesion to the type I collagen-coated substrates was not only mediated by direct binding via collagen integrins but also partly mediated by indirect binding to newly formed fibronectin fibrils that were adhering to the collagen. In addition to the observations reported in this study that showed fibronectin fibril deposition on both monomeric collagen and aligned collagen fibrils, there are several other studies in the literature that support this hypothesis. For example, there are several reports that type I collagen has binding sites for fibronectin [16,80,81,82,83], fibronectin fibril formation can occur in the absence [82,84] and presence of collagen [83,85], and that the presence of collagen fibrils induces the formation of co-localized fibronectin fibrils [84,86]. Moreover, fibronectin is secreted by corneal keratocytes during the early stages of wound healing [8]. Other studies have reported fibronectin secretion when corneal fibroblasts are cultured in 3D fibrin matrices [85,87], in the presence of Substance P [86,88], and in response to integrin activation with MnCl_2_ [87,89]. Finally, it has been reported that HTK cells secreted fibronectin in order to adhere to 3D fibrin matrices using the α_5_ integrin [85,87].

## 4. Methods

### 4.1. Preparation of Microfluidic Devices and PDMS-Coated Glass Coverslips

PDMS-coated glass coverslips and microfluidic channels were prepared as described previously [46]. Briefly, Sylgard 184 Silicon Elastomer base and curing agent (Dow Corning; Midland, MI, USA) were mixed at a mass ratio of 10:1, defoamed, and poured over photoresist templates. After curing in a PDMS oven at 80 °C for 1 h, the PDMS microchannels were cut out. PDMS-coated glass coverslips were prepared by spin coating the degassed PDMS onto 45 × 50 mm #1 glass coverslips (Brain Research Laboratories; Waban, MA, USA) and cured overnight in a PDMS oven at 80 °C.

### 4.2. Preparation of Pluronic Solution

Solutions of Pluronic were prepared as described previously [46]. A 0.2% Pluronic F-127 solution in 1X Phosphate-Buffered Saline (PBS) was used with the substrates patterned with aligned collagen fibrils, while a 1% Pluronic F-108 solution in 1X PBS was used for the monomer and fibronectin micropatterns.

### 4.3. Micropatterning and Imaging of Aligned Collagen Fibrils

Aligned collagen fibrils were deposited on PDMS-coated glass coverslips by perfusing solutions of type I collagen at defined shear rates and widths (50 μm, 100 μm, 250 μm, and 750 μm), as previously described [44,46]. Briefly, after reversibly bonding the PDMS microchannel of the desired width on the PDMS-coated glass coverslips, chilled solutions of 3 mg/mL Bovine Collagen Type I (Advanced BioMatrix; Carlsbad, CA, USA), 0.1M NaOH, and 10X Minimal Essential Media (MEM, Life Tech; Carlsbad, CA, USA) were mixed in a ratio of 8:1:1 to produce a final collagen concentration of 1.6 mg/mL. The pH was then adjusted to 7.56 by the addition of 0.1M NaOH, and infusion of the collagen solution was performed immediately in a cold room (T = 4 °C). Throughout the collagen infusion, the PDMS stamp coverslip assembly was placed on a hot plate set to 40 °C to induce polymerization of the collagen and formation of the aligned collagen fibrils. Following deposition of the aligned collagen fibrils, the microfluidic devices were carefully delaminated from the PDMS-coated glass coverslips, washed with a stream of Millipore water, and dried on the hot plate for 30 min at 40 °C.

Differential Interference Contrast (DIC) Imaging of the aligned collagen fibrils was performed with a Zeiss AxioObserver Z1 inverted microscope equipped with an Orca Flash 4.0 monochrome camera (Hamamatsu Photonics, Hamamatsu, Japan) using a 20X Plan Apochromat objective (NA = 0.75). Quantification of collagen fibril alignment was performed using the Directionality plugin in the ImageJ software (Fiji; https://imagej.net/software/fiji/downloads) as previously described [44,45,46]. The Directionality plugin computes a histogram of the alignment of the fibrils along radial angles from −90 degrees to +90 degrees. A well-defined narrow histogram with a peak at 0° suggests a high degree of fibril alignment since the substrates were imaged with the channels oriented horizontally, while histograms with broad distributions and no peak correlate with substrates that had no preferred fibril orientation. Substrates coated with random fibrils were prepared as outlined previously [46].

### 4.4. Preparation and Imaging of Other ECM Coatings

Protein micropatterns of FITC-tagged collagen monomers (Sigma Aldrich, St. Louis, MO, USA) and rhodamine/FITC-tagged fibronectin (Fischer Scientific, Waltham, MA, USA) were deposited by withdrawing the solutions through microchannels of varying widths (w = 50–750 μm) and constant height (h = 45 μm). Monomeric collagen solution was made by neutralizing 50 μg/mL FITC collagen with 0.1M NaOH. Fluorescent rhodamine-tagged fibronectin solutions were made by dilution with 1X PBS to a final solution concentration of 15 μg/mL. The collagen and fibronectin solutions were manually withdrawn into the microchannels using a syringe and allowed to statically adsorb for 2 h at room temperature. Next, the microchannels were delaminated from the PDMS-coated glass coverslips, gently washed with 1X PBS, before fluorescent images were acquired on a Zeiss AxioObserver Z1(Carl Zeiss, Thornwood, NY, USA) inverted microscope equipped with an Orca Flash 4.0 monochrome camera (Hamamatsu, Hamamatsu, Japan) using a 20X Apochromat Objective.

In some experiments, homogenous protein coatings of collage, fibronectin, or poly-L-lysine were prepared. Homogenous collagen and fibronectin-coated surfaces were prepared by adding 1 mL of FITC–collagen (50 μg/mL) solution or 1 mL rhodamine/FITC–fibronectin (15 μg/mL) solution to a PDMS ring bonded on the PDMS-coated glass coverslips [44]. The protein was allowed to adsorb for 2 h at room temperature, gently washed with 1X PBS, and dried before culturing cells. For coating with poly-L-lysine (PLL), substrates were coated with 1ml of PLL solution (Sigma-Aldrich, P8920, Saint Louis, MO) for 30 min. After 30 min, the solution was removed, and substrates were allowed to dry completely for 30 min before culturing cells.

### 4.5. Creating Protein and Cell-Repellent Regions Using Pluronic

Protein and cell-repellent regions were created on the PDMS surface using a Pluronic coating [46]. Prior to cell culture, the protein micropatterned substrates were sterilized by ultraviolet (UV) light treatment for 15 min. Next, the aligned fibril substrates were coated with 0.2% F-127 Pluronic solution or the fluorescent collagen monomer and fibronectin micropatterned substrates were coated with 1% F-108 Pluronic solution at room temperature. After incubating for 2 h, the Pluronic solutions were completely aspirated before placing the Pluronic-treated substrates in a vacuum desiccator for 30 min to allow for complete drying.

### 4.6. Cell Culture

All cell experiments were performed with a human corneal fibroblast cell line (HTK) [90,91]. HTKs were initially cultured in serum containing medium consisting of Dulbecco’s modified Eagle’s medium (DMEM) supplemented with 1% PenStrep (Invitrogen; Carlsbad, CA, USA), 10% Fetal Bovine Serum (FBS), and 0.4% sterile filtered HEPES solution [46,91]. Prior to seeding HTKs on protein-coated substrates, HTKs were cultured for 2 days in serum-free media consisting of DMEM supplemented with 100 μg/mL ascorbic acid, 100 μM nonessential amino acids (Invitrogen; Carlsbad, CA, USA), 1% PenStrep, and 1% RPMI vitamin mix. Initial adhesion experiments were performed by adding 2 mL of a HTK cell suspension (15,000 cells/mL) to the PDMS microwell assembly and culturing the cells for 24 h. For experiments investigating the effects of varying cell density, we kept the volume of the HTK cell suspension constant (2 mL) and cultured the cells on 250 μm wide monomer patterns at 3 different densities: low (5000 cells/mL), medium (10,000 cells/mL), and high (15,000 cells/mL). Finally, to account for the differences in adhesive area, we conducted another set of low-density experiments in which cells were seeded at the same cell density/area for each micropattern width to minimize cell–cell contact.

### 4.7. Antibody Blocking Experiments

For experiments with blocking antibodies, HTK cells were preincubated with purified monoclonal antibodies of mouse anti-human α5 integrin-blocking antibody (P1D6, Abcam ab78614, 25 μg/mL) α_v_β_3_-integrin (LM609, Abcam ab190147, 25 μg/mL) or β_1_-integrin (P5D2, Abcam ab24693, 25 μg/mL). Adhesion assays were performed by seeding the HTK cells onto the substrates with integrin antibody-containing medium and culturing for 24 h before fixation.

### 4.8. Immunofluorescence Imaging

For HTK staining, cells were cultured on the protein-coated substrates for 24 h and fixed with a 4% paraformaldehyde (PFA) solution for 15 min at room temperature. Following fixation, HTK cells were washed three times in PBS and permeabilized for 15 min in a 0.5% Triton X-100 in PBS solution. Cells were subsequently blocked with a solution of 2% Bovine Serum Albumin Fraction V (BSA Microbiological Grade Powder, Fisher Scientific, Hampton, NH, USA) in PBS overnight at 4 °C followed by incubation with the anti-YAP1 primary antibody (sc101199; Santa Cruz Biotech, Dallas, TX, USA) at a 1:200 dilution overnight at 4 °C. After washing in PBS, the samples were incubated in Alexa Fluor 647 conjugated secondary antibody (1:200 dilution) (Invitrogen, Carlsbad, CA, USA) for 2 h at room temperature. Fluorescent imaging of cell-deposited fibronectin was accomplished by incubating the cells with the anti-Fibronectin Antibody (Sigma-Aldrich, F0791,Saint Louis, MO, USA 1:50) in 2% BSA overnight in 4 °C. Following primary antibody incubation, cells are washed 3 times in 1X PBS and incubated with secondary antibody Alexa Fluor 488 conjugated secondary antibody (1:200 dilution) (Invitrogen, Carlsbad, CA, USA) for 2 h at room temperature. The cell nuclei and actin filaments were labeled by incubation in 4′,6-diamidino-2-phenylindole (DAPI) solution (1:1000 dilution) and Alex Fluor 546 Phalloidin (1:200 dilution, Molecular Probes, Eugene, OR, USA) for 1 h. Fluorescent imaging of YAP, actin, and nuclei was performed with a Zeiss AxioObserver Z1 inverted microscope using a 20X Apochromat Objective (N.A. = 0.75), while fibronectin deposition imaging was performed with an Olympus IX83 Inverted Spinning Disk Confocal Microscope (Olympus, Waltham, MA, USA) equipped with a 100X Silicone oil Apochromat objective (NA = 1.35) and a Orca Flash 4.0 Plus SCMOS Digital Camera (Hamamatsu, Hamamatsu, Japan).

### 4.9. Cell Morphometry and Nuclear Angle Measurements

Cell and nuclear morphometric measurements of the HTK cells were performed using ImageJ, as outlined previously [46]. Briefly, fluorescent images of DAPI were thresholded into a binary image and the projected nuclear area was measured using the ‘Analyze Particle’ plugin after checking the ‘Area’ in the ‘Set Measurements’ under the ‘Analyze’ tool in ImageJ. The degree of nuclear alignment on the different substrates was determined by first fitting the nucleus with an ellipse using the ‘Fit Ellipse’ in Image J software and measuring the angle between the major axis and the horizontal axis. Next, the measured nuclear angles were used as a measure of the overall cell alignment by calculating the orientation index (OI). An OI value of 100% indicates that the nucleus was in perfect alignment with the underlying fibrils. Multiple images at different locations were acquired for each substrate, and more than 20 cells were analyzed per substrate.

### 4.10. Quantitative Analysis of YAP Localization

ImageJ was used to compute the nucleo-cytoplasmic YAP localization. To quantify the relative YAP localization, the cell and nuclei were outlined by the DAPI and F-actin staining. After calculating the cell and nuclear area, the cytoplasmic area was computed by subtracting the nuclear area from the total cell area. The nuclear ROI obtained from the DAPI channel was overlayed on the YAP fluorescent image, and the mean YAP fluorescence in the nucleus was obtained. Similarly, the mean YAP fluorescence in the cell was obtained by overlaying the cell ROI from the fluorescent F-actin channel. The following equations were then used to obtain the cytoplasmic YAP localization:

(1) Total YAP Fluorescence in the Cell=Mean YAP Fluorescence in Cell x Cell Area

(2) Total YAP Fluorescence in the Nucleus=Mean YAP Fluorescence in Nucleus x Nuclear Area

(3) $$Total\;YAP\;Fluorescence\;in\;the\;Cytoplasm=Equation\left(1\right)-Equation\;(2)$$

(4) $$Mean\;YAP\;Fluorescence\;in\;the\;Cytoplasm=\frac{Total\;YAP\;Fluorescence\;in\;Cytoplasm}{Cytoplasm\;Area}$$

The YAP Nucleus to Cytoplasm ratio was then calculated using

(5) $$\frac{N}{C}Ratio=\frac{Mean\;YAP\;Fluorescence\;in\;Nucleus}{Mean\;YAP\;Fluorescence\;in\;Cytoplasm}$$

### 4.11. Quantitative Analysis of Fibronectin Deposition

Fibronectin matrix secretion quantification was performed as outlined previously [89,90]. ImageJ was used to quantify the fibrillar secretions. Specifically, to quantify the fibronectin secretion, background subtraction was used to eliminate the background, followed by the analyze particle plugin.

### 4.12. Statistical Analysis

All the calculations, graphing, and statistical analyses of the experimental data were performed using GraphPad Prism software version 8.4.3 (GraphPad Software, San Diego, CA, USA). Unless otherwise noted, data were represented as mean ± standard deviation. When appropriate, data were analyzed by a one-way or two-way ANOVA followed by Tukey’s post hoc test. For all analyses, *p* < 0.05 was considered statistically significant.

## 5. Conclusions

Prior studies have shown that both biochemical (i.e., ECM composition, growth factors, proteoglycans) and biophysical (i.e., topography, ECM elasticity, cell–cell contact) cues can impact corneal fibroblast behavior. How corneal fibroblasts integrate simultaneous exposure to these various cues is not well understood, but it is important in developing new therapies to reduce corneal scarring. Taking advantage of our ability to fabricate micropatterns of ECM proteins with different compositions, topographies, and geometries, we were able to begin to untangle the relative importance of these cues in YAP-mediated signaling in corneal fibroblasts. In particular, we showed the importance of initial cell seeding density on cell alignment, cell crowding, and YAP localization on various ECM coatings. We demonstrated that the composition (e.g., collagen vs. fibronectin) and topography (e.g., flat, random, or aligned) of the ECM also modulate YAP localization as well as the secretion of fibrillar fibronectin. We provided new data on how specific integrin subunits modulate YAP localization on different ECM proteins. Given the important role that YAP plays in contact inhibition, mechanotransduction, and TGFB signaling pathways in cornea keratocytes and fibroblasts, we believe that the cell seeding and integrin blocking results will be beneficial in informing future studies on novel corneal scaffolds in terms of cell seeding densities and scaffold composition. Based on the success of aligned synthetic substrates to promote the differentiation of human corneal stromal stem cells (hCSSCs) into functional keratocytes [90], we also envision that the topography and geometry of the aligned collagen fibril micropatterns in this study, which utilized type I collagen (i.e., the major ECM protein of the cornea stroma), may be useful in developing collagen-based constructs for corneal repair and regeneration by combining them with hCSSCs. Finally, the results of the integrin-blocking experiments might lead to the identification of novel therapeutic targets.

## Data Availability

Data is contained within the article and Supplementary Materials.

## Supplementary Materials

The following supporting information can be downloaded at: https://www.mdpi.com/article/10.3390/ijms26031183/s1.
