# Supplementary material for: Effects of Cell Seeding Density, Extracellular Matrix Composition, and Geometry on Yes-Associated Protein Translocation in Corneal Fibroblasts
Source: Int J Mol Sci. 2025 Jan 29;26(3):1183. doi: 10.3390/ijms26031183 (PMC11818043; doi:10.3390/ijms26031183)
Supplement: Supplementary file 1 [file ijms-26-01183-s001.zip › ijms-3357496-supplementary.pdf]

Supplementary Data

for

Effects of Cell Seeding Density, ECM Composition, and Geometry on Corneal Cell Behavior

Divya Subramanian[1], Nathaniel S. Tjahjono[1], Satweka Nammi[1], Miguel Miron-Mendoza[2], Victor D. Varner[1,3], W. Matthew Petroll[2,3], and David W. Schmidtke[1,3,*]

[1] Department of Bioengineering, University of Texas at Dallas, Richardson, TX;
[2] Department of Ophthalmology, University of Texas Southwestern Medical Center at Dallas, TX
[3] Department of Biomedical Engineering, University of Texas Southwestern Medical Center at Dallas, TX

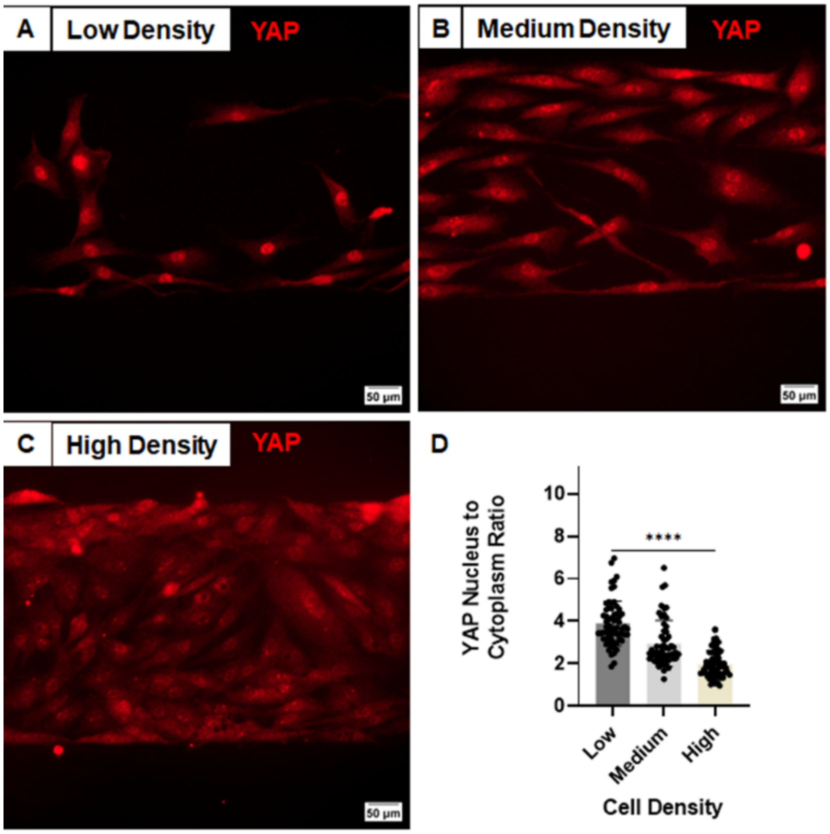

Supplementary Figure S1: YAP Localization is regulated by cell density: YAP localization shifts from nuclear (low) to cytoplasmic YAP (high). Data represent Mean ± Standard Deviation over 4 repeats.

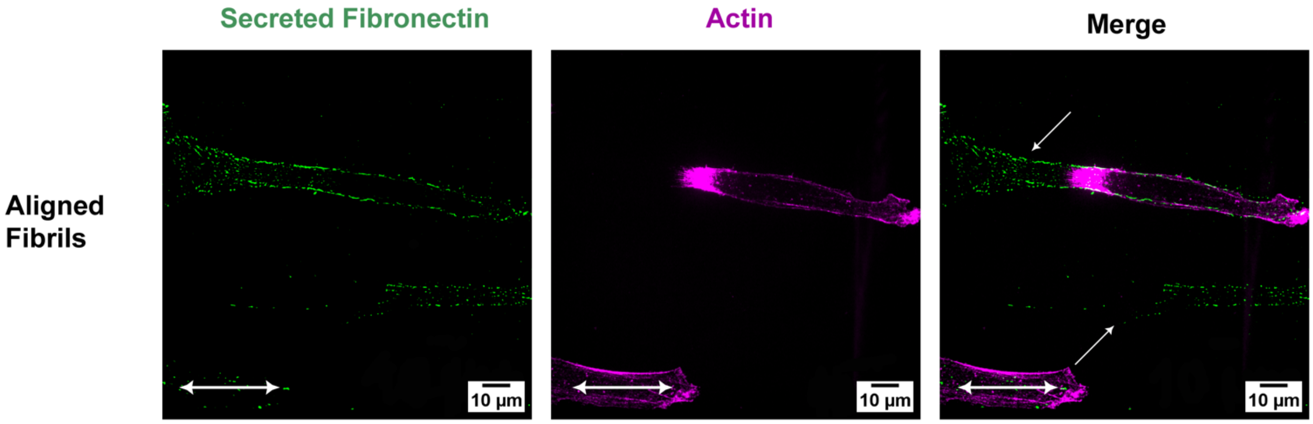

Supplementary Figure S2: Fibronectin tracks are observed in close proximity to the cell cytoskeleton: Immunofluorescence Confocal Images for Fibronectin tracks (white arrows) observed in close proximity to the cell membrane. Cells were labeled for Fibronectin (Green) and Actin (Magenta). Scale Bar = 10 µm. Horizontal lines indicate aligned fibril direction

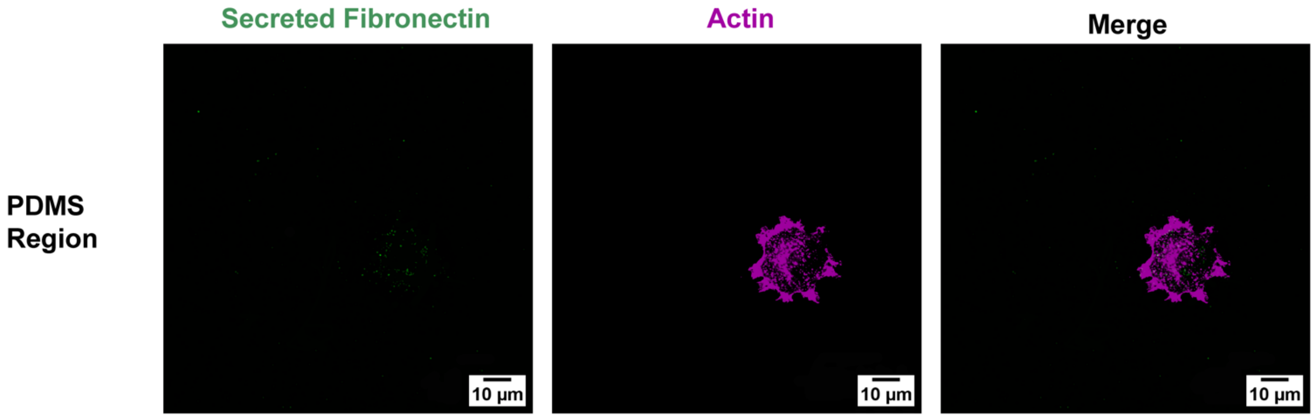

Supplemental Figure S3: HTK Adhesion to Uncoated PDMS did not Stimulate Fibronectin Deposition: Immunofluorescence Confocal Images for Fibronectin deposition by the cells on PDMS regions. Cells were labeled for Fibronectin (Green) and Actin (Magenta).

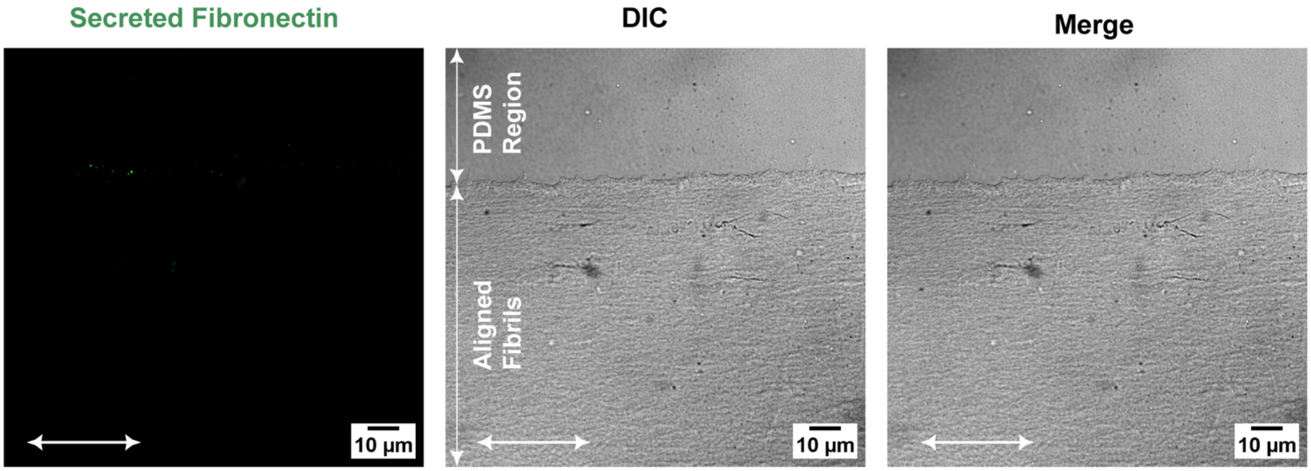

Supplementary Figure S4: No fibronectin staining was observed in absence of cells: Immunofluorescence Confocal Images for Fibronectin deposition without any cells. Substrates were labeled for Fibronectin (Green). Dashed horizontal lines indicate fibril direction (L-R) Scale Bar = 10 µm
